# Supplementary material for: Implementation of mental health policies and plans across the WHO European region: Barriers and facilitators
Source: Glob Ment Health (Camb). 2025 Oct 22;12:e121. doi: 10.1017/gmh.2025.10070 (PMC12641295; doi:10.1017/gmh.2025.10070)
Supplement: Guerrero et al. supplementary material [file S2054425125100708sup001.docx]

# Appendix

**Fig. 1: Key informant interview guide flow-diagram - implementation**


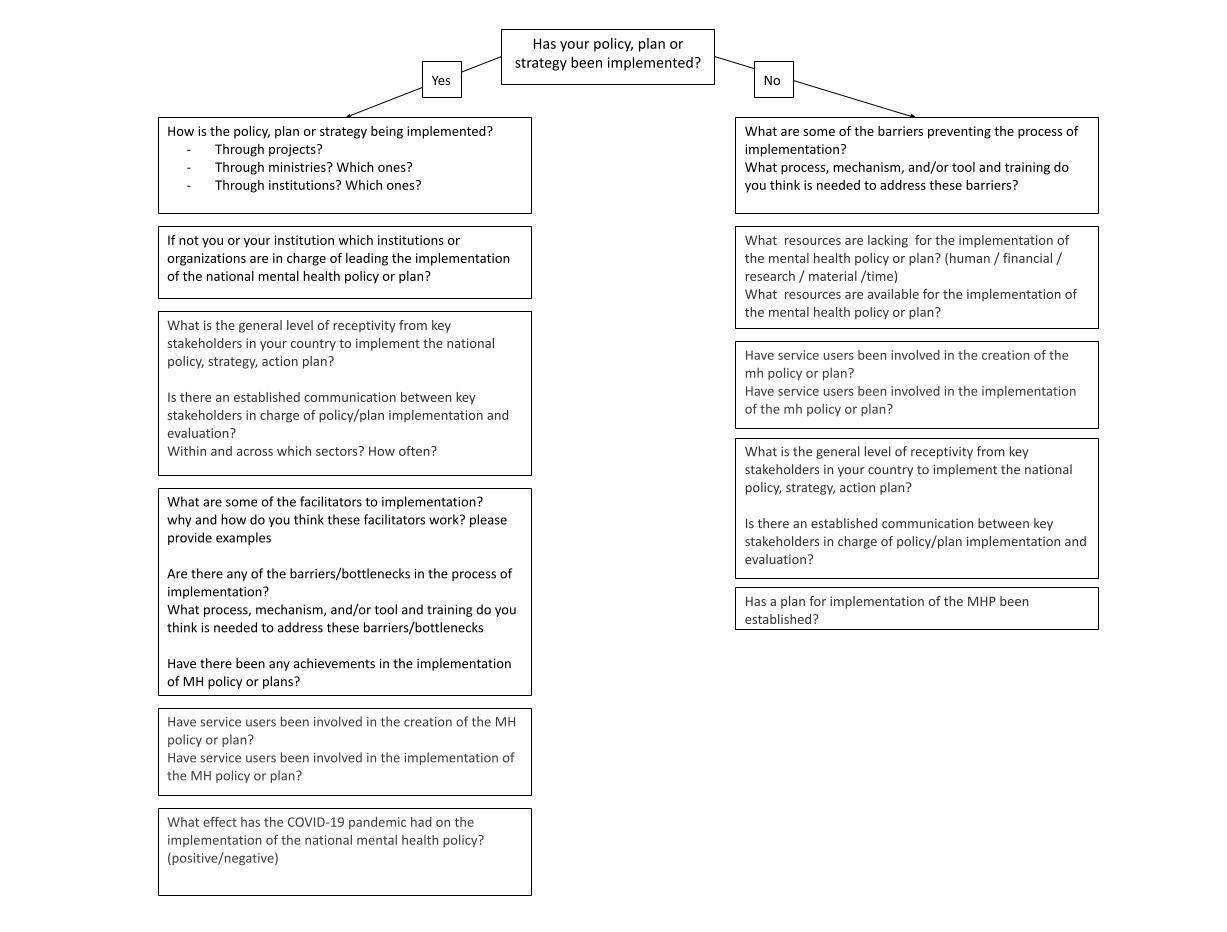


**Fig. 2: Key informant interview guide flow-diagram - evaluation**


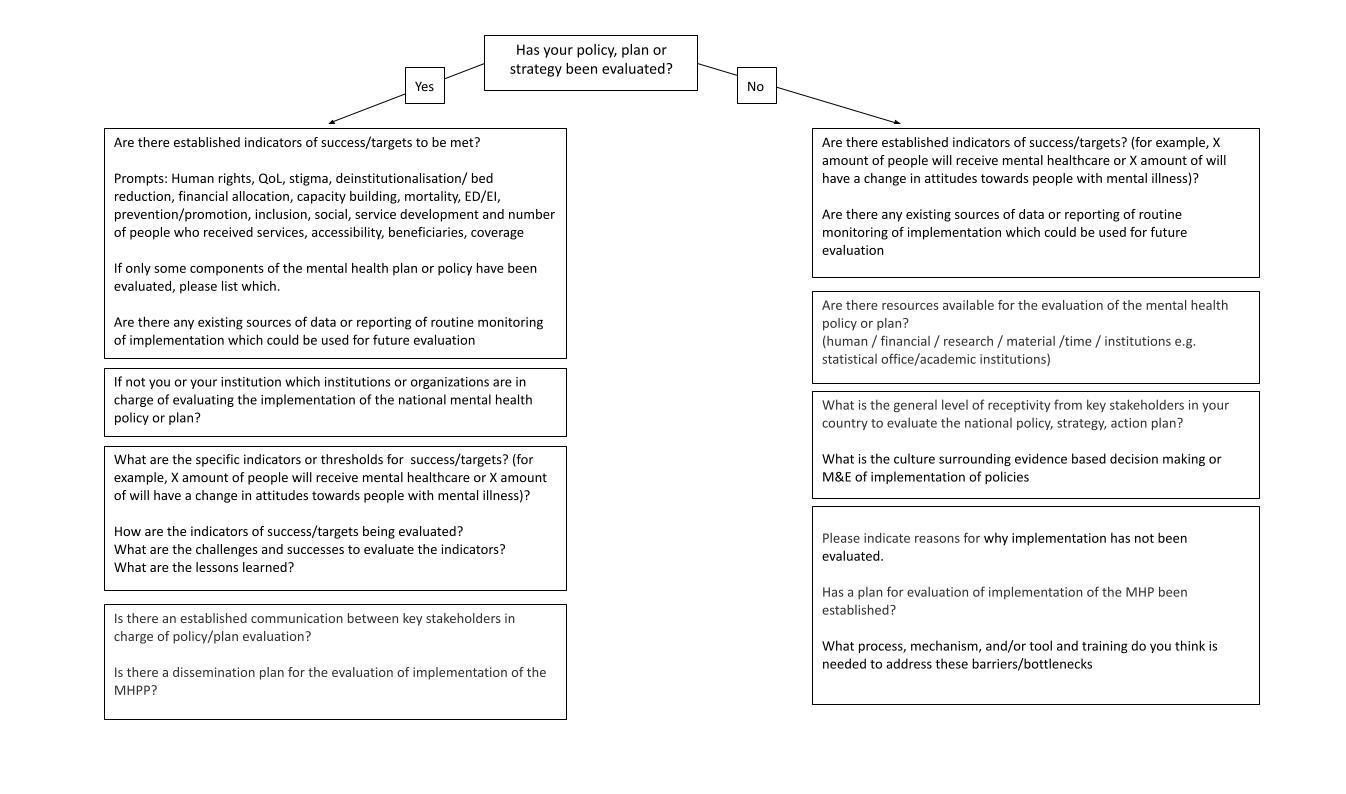


**Table 1: Frequencies of codes in themes from key informant interviews**

| Theme | Frequency (n) | Frequency (%) |
| --- | --- | --- |
| Implementation system | 75 | 18.3% |
| Implementation status | 7 | 1.7% |
| Communication | 47 | 11.5% |
| Collaboration and cooperation | 69 | 16.9% |
| Stakeholder receptivity | 42 | 10.3% |
| Reform of mental healthcare | 11 | 2.7% |
| Resources | 105 | 25.7% |
| Achievements | 53 | 13% |
| Total | 409 |  |
